# The immediate effects of kinesiology taping on cutaneous blood flow in healthy humans under resting conditions: A randomised controlled repeated-measures laboratory study

**DOI:** 10.1371/journal.pone.0229386

**Published:** 2020-02-21

**Authors:** Gourav Banerjee, Michelle Briggs, Mark I. Johnson

**Affiliations:** 1 Centre for Pain Research, School of Clinical and Applied Sciences, Leeds Beckett University, Leeds, England, United Kingdom; 2 Division of Nursing, Midwifery and Social Work, School of Health Sciences, Faculty of Biology, Medicine and Health, University of Manchester, Manchester Academic Health Science Centre, Manchester, England, United Kingdom; Anglia Ruskin University UK, UNITED KINGDOM

## Abstract

**Background:**

Kinesiology taping (KT) is used in musculoskeletal practice for preventive and rehabilitative purposes. It is claimed that KT improves blood flow in the microcirculation by creating skin convolutions and that this reduces swelling and facilitates healing of musculoskeletal injuries. There is a paucity of physiological studies evaluating the effect of KT on cutaneous blood microcirculation.

**Objectives:**

The purpose of this parallel-group controlled laboratory repeated measures design study was to evaluate the effects of KT on cutaneous blood microcirculation in healthy human adults using a dual wavelength (infrared and visible-red) laser Doppler Imaging (LDI) system. KT was compared with rigid taping and no taping controls to isolate the effects associated with the elasticity of KT

**Methods:**

Forty-five healthy male and female human adults were allocated to one of the three interventions using constrained randomisation following the pre-intervention measurement: (i) KT (ii) ST (standard taping) (iii) NT (no taping). Cutaneous blood perfusion was measured using LDI in the ventral surface of forearm at pre-intervention, during-intervention and post-intervention in a normothermic environment at resting conditions.

**Results:**

Mixed ANOVA of both infrared and visible-red datasets revealed no statistically significant interaction between Intervention and Time. There was statistically significant main effect for Time but not Intervention.

**Conclusion:**

KT does not increase cutaneous blood microcirculation in healthy human adults under resting physiological conditions in a normothermic environment. On the contrary, evidence suggests that taping, regardless of the elasticity in the tape, is associated with immediate reductions in cutaneous blood flow.

## Introduction

Kinesiology taping is a popular therapy amongst musculoskeletal professionals including physical and sports therapists, chiropractors, osteopaths and athletic trainers for preventing and rehabilitating musculoskeletal injuries and improving sports-related performance. Conventional taping and strapping techniques use rigid or minimally elastic adhesive tapes and strapping materials to provide compression, immobilisation and support to the injured soft tissues and joints aimed at promoting recovery. In contrast, kinesiology taping uses polymer elastic cotton-based water-resistant adhesive (kinesiology) tape that can be stretched longitudinally up to 60% or more of its resting length and worn continuously for 3–5 days to support soft tissues and joints whilst not restricting movements, thereby allowing physical and sports-related activities such as running and swimming [1–5].

**Fig 1:**
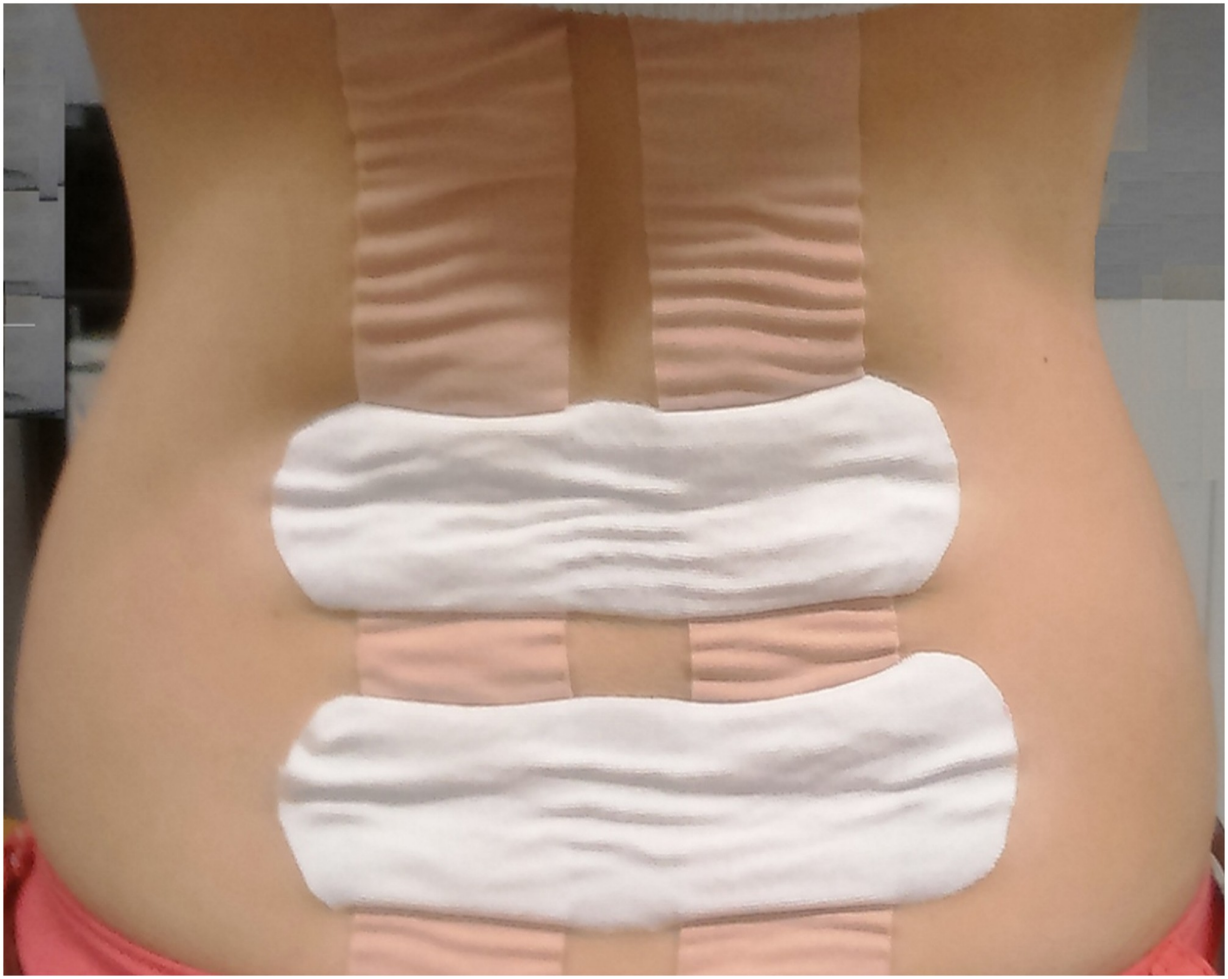
Figure showing convolutions of skin upon kinesiology taping.

The scope of the use of kinesiology taping in healthcare has extended beyond musculoskeletal practice to include management of cancer-related lymphedema [6–8]. It is claimed by the advocates that kinesiology taping creates convolutions of the skin causing the epidermis to lift away from the underlying tissues and the resultant decompression alters flow of blood and lymph in the microcirculation, which in turn might reduce swelling [2–4]. The human dermis encompasses interwoven network of blood and lymphatic vessels functionally involved in thermoregulation, meeting the nutritional and metabolic demands of the skin, and redistributing blood flow during stress. The flow of blood in the single-layered endothelial capillaries depends on vasomotion controlled by dynamic multiple interacting mechanisms that are influenced by local, neurogenic, metabolic, endothelial and myogenic factors [9–13], and during inflammation the endothelial vessels become enlarged [14,15]. Kinesiology taping can be applied to produce visible convolutions of skin (Fig 1), so it seems plausible that mechanical deformation of skin could lift the epidermis from the dermis increasing interstitial volume and decreasing interstitial pressure, altering the flow of interstitial fluid, blood and lymph in the microcirculation of skin and superficial tissues [16]. There is radiological evidence that kinesiology taping produces mechanical deformation of tissues underneath the tape in humans [17,18]. Evidence from animal models suggests that this leads to an increase in epidermal-dermal distance resulting in increased lymph flow [19,20]. It is known that the flow of interstitial fluid is critically important in the function and pathogenesis of tissues [21–23] and is of therapeutic significance with important clinical implications in pain management and musculoskeletal rehabilitation [24,25].

There is a paucity of studies on humans to determine whether deformation of tissues beneath kinesiology taping produces alterations in cutaneous blood microcirculation. A randomised placebo-controlled clinical trial (RCT) on 120 postmenopausal women with mild chronic venous insufficiency found that kinesiology taping increased peripheral venous flow in the gastrocnemius muscle measured by photoplethysmography with a concurrent decrease in swelling [26]. Studies on healthy human participants are often used as a precursor to clinical trials as they provide information about the effect of interventions on normal physiology in the absence of pathology. In contrast to the findings of this RCT [26], laboratory studies using laser Doppler flowmetry (LDF) imaging techniques found that kinesiology taping did not increase cutaneous [27,28] or skeletal muscle [29] blood flow when measured under a variety of experimental conditions (for e.g., at rest and during exercise). It has been recognised that there are methodological challenges in measuring cutaneous blood microcirculation [30] including controlling of extraneous variables such as posture [31], laboratory lighting conditions [32] and blood flow measurement techniques. For example, previous laboratory studies [27–29] were criticised because LDF techniques use a stationary probe in contact with the skin that can only measure blood perfusion from a discrete area of skin (1 to 2 mm^3^) and therefore LDF may miss variations in microcirculation in adjacent areas of the skin [33]. Furthermore, the contact probe used during LDF may deform the skin introducing measurement artefact and reductions in the spatial resolution of the measurement [30,34–37].

Laser Doppler imaging (LDI) is a modern technique used to measure blood perfusion of the skin. LDI uses a small (for e.g., 10 mm diameter) fibre-optic laser probe consisting of illuminating fibres that emit laser into the tissues and detecting fibres that detect the back-scattered photons enabling continuous blood perfusion measurement [38]. LDI is considered superior to LDF because it measures microcirculation using non-contact scanning of the skin and this enables measurements to be taken over larger areas. Measurements using LDI have higher reliability and spatial resolution compared with LDF [34,37,39–41]. LDI is considered the gold standard for assessing blood flow in various types of skin wound and is recommended by the National Institute for Health and Care Excellence [42,43]. The purpose of this study was to evaluate and compare the effects of kinesiology taping to standard rigid taping and no taping controls on cutaneous blood microcirculation in healthy human adults using LDI under resting conditions in a normothermic environment (H_0_: no significant mean difference in change in blood perfusion amongst the three taping conditions). Kinesiology taping was compared with standard rigid taping and no taping controls to isolate the effects associated with the elasticity of kinesiology tape.

## Materials & methods

### Study design

This was a repeated measures parallel-group controlled laboratory study where cutaneous blood perfusion (mm/sec flux) was measured using LDI before, during and after one of three possible interventions: (i) Kinesiology taping (using RockTape, a proprietary kinesiology tape); (ii) Standard taping (using BSN medical Strappal^®^ tape, a proprietary rigid adhesive tape); (iii) No taping. This study was approved by the Research Ethics Committee of Leeds Beckett University (reference number 14366).

### Sample size

Sample size was calculated in G*power software v 3.1.9.2 [44] using conservative estimates of small effect size of 0.25, 95% power, 5% Type 1 error, and assumptions of repeated measures within-between interaction ANOVA with six measurements and three groups. The total sample size was estimated to be 36 participants with 12 participants per group (see S1 Fig). It was decided to recruit 15 participants per group to account for attrition and allow for the possibility of removal of outliers in the dataset for sensitivity analysis.

### Participant enrolment

Healthy participants were recruited by poster advertisements in Leeds Beckett University. Interested volunteers received a participant information pack including information on eligibility to participate in the study. The exclusion criteria were: (i) under the age of 18 years; (ii) known skin sensitivity (e.g., allergy to adhesive tapes); (iii) known allergy to low intensity laser light; (iv) present history of medical illness; (v) previous history of injury or surgery of the left forearm, (vi) scar in the ventral surface of left forearm; (vii) current intake of prescribed or over-the-counter medication; (viii) pregnancy; and (ix) unable to comprehend simple instructions in English language. There was no restriction on sex/gender, upper age, ethnicity, nor body mass index, although this was recorded. Volunteers were asked to refrain from moderate to vigorous exercise and not to consume products containing caffeine and alcohol at least 6 hours prior to the experiment [45]. Volunteers with tattoos on the ventral surface of the forearm were assessed for suitability for taping and whether it would be possible to undertake an LDI of non-tattooed skin on a case-by-case basis. Prior to signing the informed-consent form, all eligible volunteers were provided with a detailed explanation of the study including the advice that they could withdraw consent at any time and without giving a reason. After signing consent, participants provided demographic data (Table 1). Ethnicity was categorised according to the recommended ethnic group survey in England [46]. No incentive or compensation was offered for participating in this experiment.

**Table 1:** Demographic characteristics and anthropometric data of all participants.

Variable		Total	NT	ST	KT
**Sex (n)**	Male	12	4	2	6
	Female	33	11	13	9
**Age (years) (**mean±SD)		21.1±3.9	20.8±4.5	20.3±2.4	22.1±4.5
**BMI (kg/m**^**2**^**) (**mean±SD)		24.3±3.7	24.8±3.8	23.1±2.6	25.0±4.4
**Ethnicity (n)**	White British	31	10	12	9
	Other White Background	5	2	2	1
	White & Black Caribbean	2	1	0	1
	White & Asian	3	0	1	2
	Indian	1	1	0	0
	Arab	3	1	0	2

Abbreviations: NT, no taping; ST, standard taping; KT, kinesiology taping

### Experimental procedure

**Fig 2:**
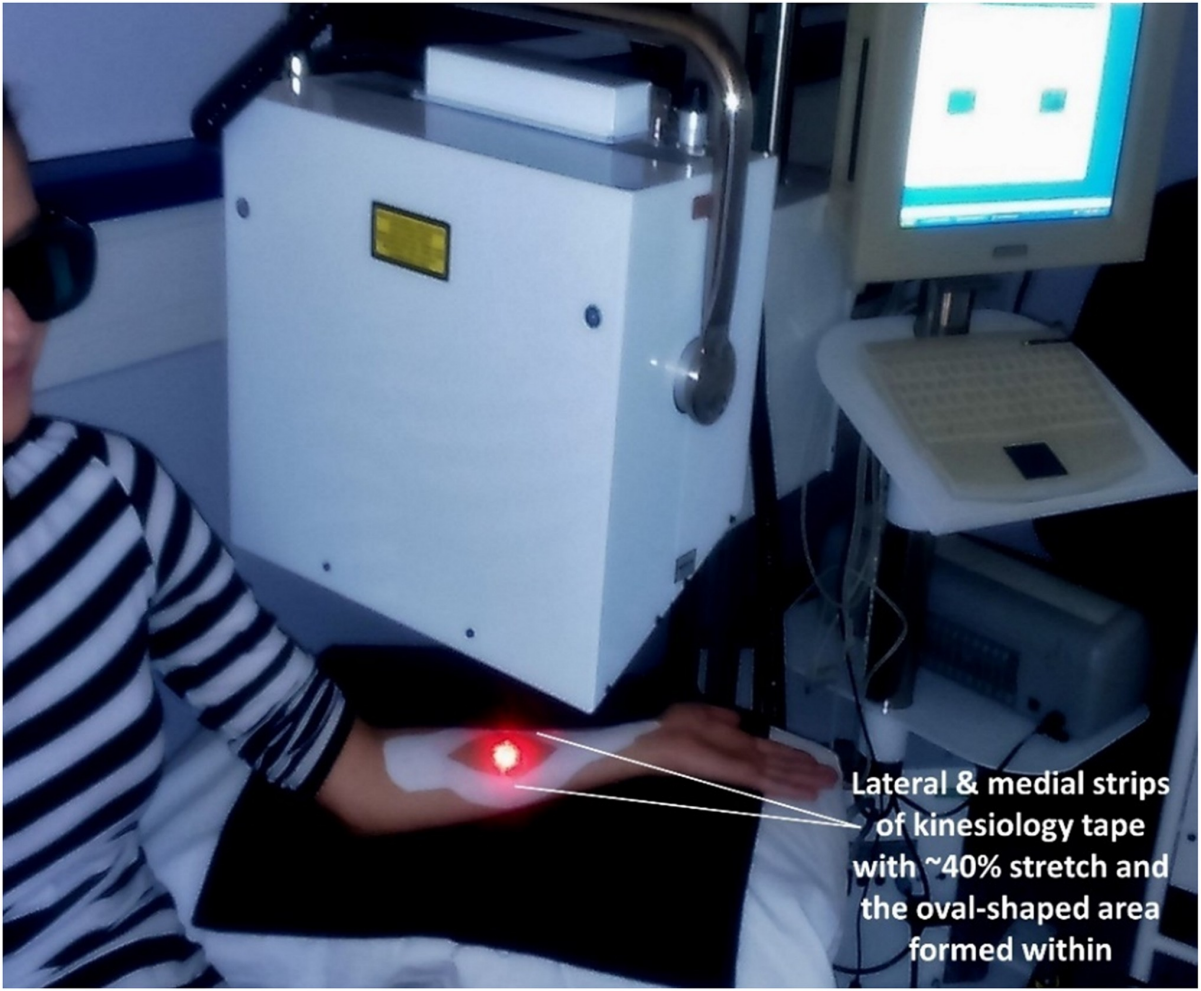
Demonstration of laser Doppler imaging of the oval-shaped site of testing formed by the inner margins of lateral and medial strips of tape.

**Fig 3:**
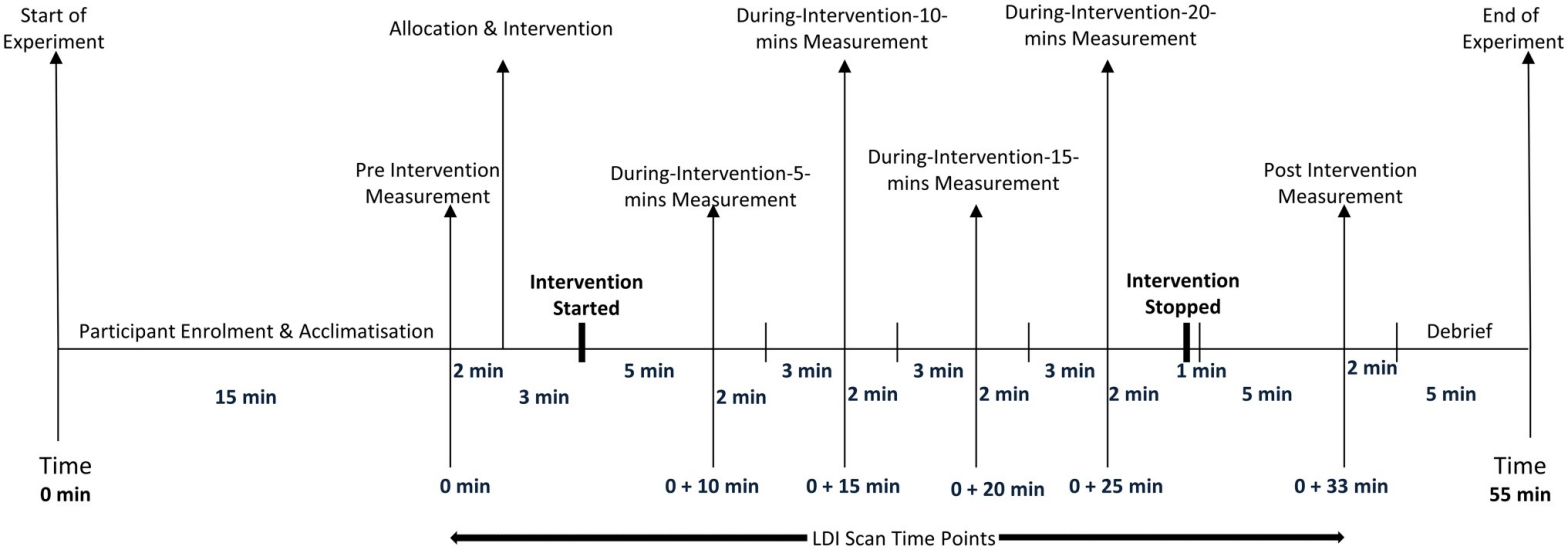
Sequence of events of the experimental procedure and laser Doppler imaging scan time points.

Each participant attended the Pain and Rehabilitation laboratory, Leeds Beckett University for one experimental visit lasting no longer than 90 minutes. The time taken for enrolment (15-minutes) enabled participants to acclimatise to the laboratory environment and attain a rested state [32,47]. The laboratory environment was low-level lighting to avoid interference with the laser Doppler with the ambient temperature recorded between 22°C-24°C during experimental sessions [30,32,48,49]. Measurements of cutaneous blood perfusion were taken with participants sitting comfortably in a long sitting posture on a plinth positioned adjacent to the LDI (Fig 2). Measurements of cutaneous blood perfusion were taken before the intervention (1x pre-intervention), with the intervention applied (4 x during-intervention with the tape in situ), and then after the intervention removed (1 x post-intervention after the removal of tape) (Fig 3). Each measurement scan lasted 2 minutes and 2 seconds. The individual in this manuscript (Fig 2) has given written informed consent (as outlined in PLOS consent form) to publish these case details.

### Randomisation and blinding

Participants were randomised to one of the three intervention groups immediately after the pre-intervention measurement. Constrained block randomisation was used whereby participants selected one of three opaque envelopes that contained a random number generated using a computer software that was associated with a particular intervention. The process of allocation was concealed, however, once the sequence number was revealed, the principal investigator (GB) became aware of the group allocation and subsequently assumed the roles of therapist, data collector and analyst. The participants allocated to the taping groups were able to see the taping interventions, they, however, were naïve to the hypothesis of study and the taping procedures.

### Measurement of cutaneous blood perfusion

A dual wavelength LDI system comprising of visible-red (633 nm) and infrared (830 nm) wavelengths (Fig 2) (MoorLDI2-2λ^™^, Moor Instruments Ltd., Devon, UK) was used to measure cutaneous blood perfusion at the ventral surface of the forearm. The principles of LDI have been described previously [38,42,48,50,51]. Briefly, a monochromatic low-intensity coaxial laser beam is transmitted into an area of the skin to a depth of approximately 0.5 to 2 mm. Red blood cells moving in microvasculature cause changes in the magnitude and frequency distribution of wavelength of the laser light and this is photo-detected by the LDI equipment. This Doppler shift to laser light is processed and a two-dimensional colour-coded image is created to represent the number and velocity of moving red blood cells in the microcirculation of the scanned area (i.e., flux and expressed in arbitrary perfusion units).

The scan area for imaging was 2.8 cm x 2.8 cm (96 x 96 pixels, scan time 10 ms/pixel) and centred in the oval-shaped area of exposed skin created by the inside margins of the taping intervention (Fig 2). The image resolution was set as 96x and 96y and general scan settings including calibration were set as per manufacturer recommendations for bandwidth, gain levels, spatial correction and background threshold with the distance between the detector and forearm tissue set at 23 cm. Infrared (IR) and visible-red (VR) wavelengths were used to simultaneously measure cutaneous blood microcirculation at depths ranging from approximately 0.5 mm to 2 mm. VR wavelength measures superficial blood microcirculation as there is more energy loss due to the skin barrier. IR wavelength penetrates deeper into the tissues thus minimising any effects of skin pigmentation [35,38,52].

The ventral surface of the forearm was chosen as the site to measure cutaneous blood perfusion because the area is generally devoid of hair follicles. This helps to minimise LDI measurement artefacts [32] is convenient to access for imaging and taping, and is one of the preferred sites for assessing cutaneous endothelial function using LDI technologies [53–55]. Before the start of each scan, the forearm was positioned with the laser beam fixated on a point 10 cm (ventral surface forearm) below the centre of cubital fossa. The point was drawn to minimise spatial variation between scans [30]. Participants were asked to wear laser protective eyewear through the entire duration of LDI scanning, and during each scan participants were instructed to remain still, refrain from talking and to fixate their gaze on a dot on the adjacent wall [32]. Between the scan periods, participants remained seated and relaxed and were instructed not to move their measurement arm (other than slight movements of the forearm and wrist) as a change in posture is known to cause changes in microcirculation [31]. The arm was repositioned prior to each scan.

### Taping interventions

Kinesiology tape (RockTape) and standard tape (BSN medical Strappal^®^) were matched for shape (rectangle), length (20 cm), width (5 cm) and colour (plain white) and pre-cut into Y-shape, i.e., from a point 5 cm below the upper end, the tapes were cut into two equal halves such that each of the split-halves was of 15 cm of length and 2.5 cm of width.

Kinesiology tape was applied by anchoring its upper end, i.e., 5 cm length, 5 cm width, off tension (approximately 10–15% inherent stretch after peeling from paper backing) just below the cubital fossa with the wrist dorsiflexed and the elbows fully extended to stretch soft tissues in the ventral surface of the forearm. The middle section of each of the split-halves of kinesiology tape, i.e., approximately 10 cm length, 2.5 cm width, were stretched by 4 cm to achieve approximately 40% of the original length (length after stretching = approximately 14.0 cm) and attached to the skin following the medial and lateral borders of the forearm. This formed an oval shaped area with the lower end of the tape, i.e., 5 cm length, 2.5 cm width, applied off-tension (Fig 2). A measurement tape was used to standardise the length of stretch of kinesiology taping. Procedures recommended by the manufacturers of kinesiology tape for applying the tape were followed. This included cutting/rounding the edges of the tape, applying the ends of the tape off tension and rubbing along the length of tape for approximately 10 seconds to ‘activate’ the heat-sensitive glue for optimal adherence with the skin and removing the tape in the direction of hair growth keeping close to the surface of skin [56,57]. It was decided to not clean the left ventral surface forearm skin with alcohol preparation at the start of the experiment as doing so could have altered cutaneous blood microcirculation measurements.

The standard tape was applied in an identical manner to kinesiology tape except (i) the wrist was not dorsiflexed, and the elbow not fully extended so that soft-tissues in the ventral surface of the forearm were not stretched, and (ii) there was no attempt to stretch the tape. The surface of the standard tape after application, like the kinesiology tape, was rubbed for approximately 10 seconds to account for the effect of rubbing the kinesiology tape might have on cutaneous blood microcirculation.

Participants allocated to the no taping group sat on the plinth through the rest of the experiment and had the measurements taken at the fixed time intervals (Fig 3).

### Perfusion analysis

**Fig 4:**
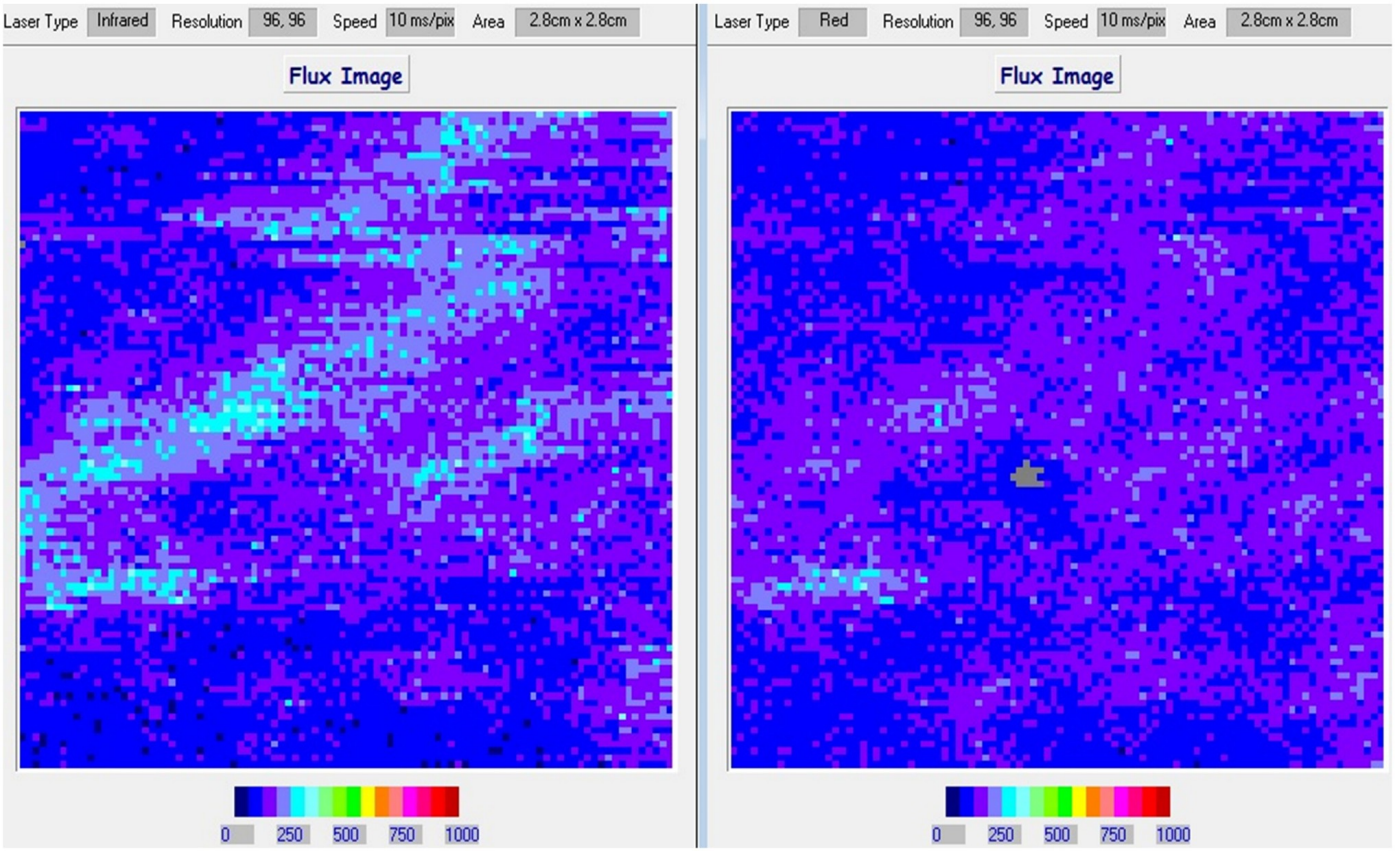
Laser Doppler imaging (LDI). An example of the infrared (left) and visible-red (right) laser blood perfusion images recorded from the site of testing of a 21-year old White British female participant at the time point during-intervention 10 minutes. The point drawn using washable marker can be seen in the centre of the visible-red image.

The spatial distribution of tissue blood perfusion was analysed using the manufacturer’s software package (MoorLDI^™^ Image Reviewer Version 5.3 D). A rectangular region of interest corresponding to the size of the laser Doppler image (i.e., 2.8 cm x 2.8 cm) was created to calculate mean ± SD blood perfusion for IR and VR for each measurement time point for each participant (Fig 4).

### Statistical analysis

**Fig 5:**
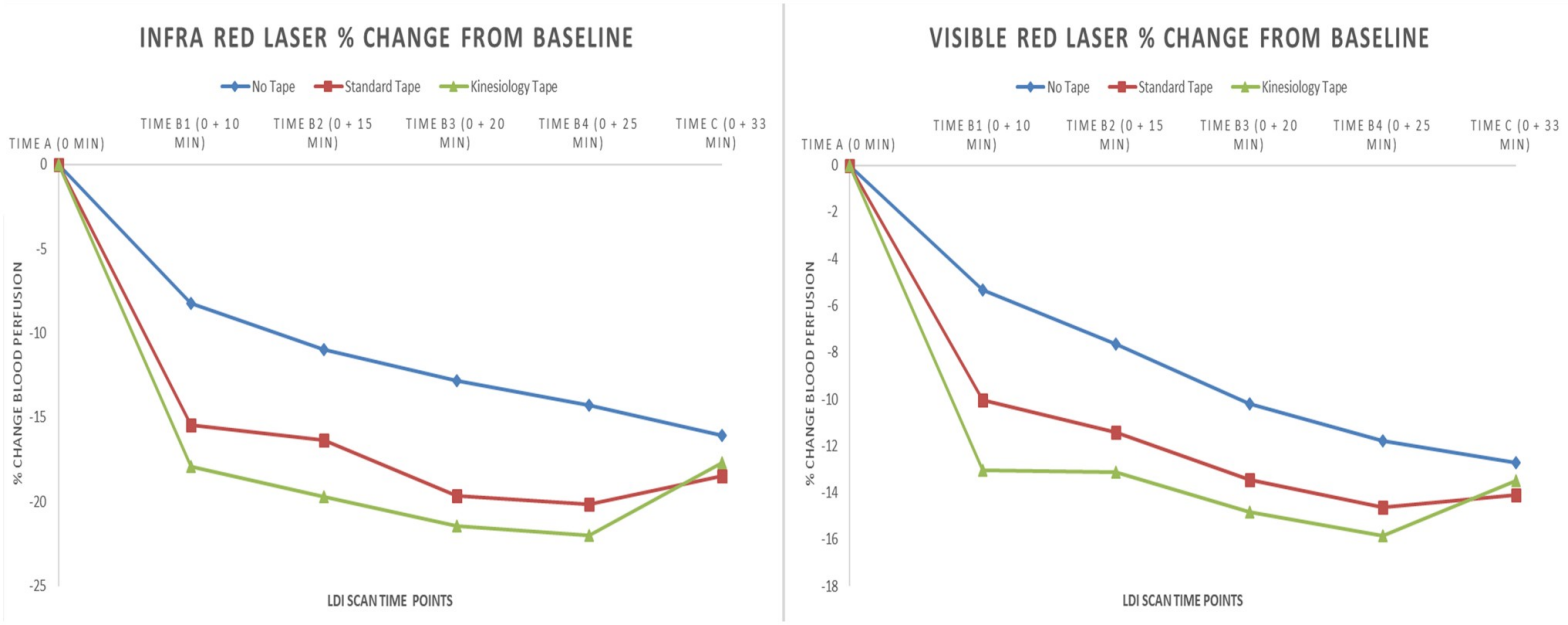
Figure showing percentage change of blood perfusion from baseline measured by infrared (left) and visible-red laser (right). Scan time point A corresponds to pre-intervention, B1 corresponds to during-intervention-5-mins, B2 corresponds to during-intervention-10-mins, B3 corresponds to during-intervention-15-mins, B4 corresponds to during-intervention-20-mins, C corresponds to post-intervention.

Data were imported to Microsoft Excel (version 2013) to calculate the mean and standard deviation of the demographic characteristics of participants and plots of blood perfusion unit against time were made for each participant and visually inspected for anomalous data. Data were then imported to SPSS for Windows (IBM Corp, 2013, version 22.0) software for statistical analyses. IR and VR datasets were tested to judge suitability for parametric analysis using mixed ANOVA [58]. Post analyses, it was decided to log10 transform both IR and VR datasets as certain critical assumptions were violated. A between-within subjects (mixed) 6 x 3 factorial analysis of variance (ANOVA) was performed on mean blood perfusion for IR and VR datasets with Time (six levels: pre, during-5mins, during-10mins, during-15mins, during-20mins, post-5mins) being the within-subject factors and Intervention (three levels: kinesiology taping, standard taping, no taping) between-subject factors. A Greenhouse–Geisser correction was used if Mauchly’s test showed that sphericity could not be assumed. Adjustments were made for multiple comparisons using the Bonferroni correction. Statistical analysis was on an intention-to-treat basis and the significance was set at *p* ≤ 0.05. Partial eta squared (η2_p_) was reported as measure of the estimate of effect size, and as a rule of thumb (Cohen), values of 0.0099, 0.0588, and 0.1379 were used to indicate small, medium and large effects, respectively. Data were analysed without subtracting the biological zero (residual flux), however, results are expressed in terms of the recommended percentage change from pre-intervention in arbitrary perfusion units [30,59]. The graphs (Fig 5) show visualisation of relative percentage changes in mean blood perfusion from the baseline across the three interventions [60]. To analyse differences in age and BMI across the three groups, one-way ANOVA was used. Distribution of sex across the groups could not be evaluated as an assumption for chi-squared test of homogeneity was violated.

## Results

### Characteristics of the sample population

Forty-five participants completed the study. A one-way ANOVA found no significant differences in age and BMI across the three groups, which suggests that randomisation was successful. Demographic characteristics and anthropometric data of the participants are presented in Table 1. Mean ± SD blood perfusion data are presented in Table 2.

**Table 2:** Group x Time mean counts of blood perfusion of infrared (IR) and visible-red (VR) data.

		Pre-intervention-		During-intervention-5-mins		During-intervention-10-mins		During-intervention-15-mins		During-intervention-20-mins		Post-intervention	
Laser	Group	Mean±SD	95% CI (LB/UB)	Mean±SD	95% CI (LB/UB)	Mean±SD	95% CI (LB/UB)	Mean±SD	95% CI (LB/UB)	Mean±SD	95% CI (LB/UB)	Mean±SD	95% CI (LB/UB)
**IR**	**NT** (n = 15)	109.61±42.29	85.37/133.85	100.57±34.66	80.70/120.43	97.56±34.53	77.77/117.35	95.53±35.77	75.02/116.03	93.96±32.87	75.12/112.80	91.98±35.10	71.86/112.10
	**ST** (n = 15)	106.23±20.74	94.75/117.72	89.81±14.38	81.84/97.77	88.85±13.67	81.27/96.42	85.34±12.58	78.38/92.30	84.84±11.37	78.54/91.14	86.59±17.87	76.69/96.48
	**KT** (n = 15)	108.76±44.20	84.28/133.24	89.29±30.12	72.61/105.97	87.32±31.01	70.15/104.49	85.45±30.87	68.35/102.54	84.81±29.45	68.50/101.12	89.50±35.78	69.69/109.31
**VR**	**NT** (n = 15)	108.39±27.20	92.80/123.98	102.60±23.52	89.12/116.08	100.11±22.47	87.23/112.99	97.35±22.99	84.17/110.53	95.61±21.18	83.47/107.76	94.60±23.37	81.21/107.99
	**ST** (n = 15)	108.29±15.97	99.45/117.13	97.41±10.00	91.87/102.95	95.92±8.69	91.11/100.73	93.71±9.32	88.54/98.87	92.43±7.95	88.02/96.83	93.02±12.10	86.32/99.72
	**KT** (n = 15)	104.13±31.49	86.69/121.57	90.53±19.80	79.57/101.50	90.49±21.26	78.71/102.26	88.68±19.07	78.12/99.24	87.65±19.84	76.66/98.63	90.09±20.84	78.55/101.63

Abbreviations: n, number of participants per group (NB: cutaneous blood perfusion of each participant was measured simultaneously by IR and VR, i.e., in total, n = 45); NT, no tape; ST, standard taping; KT, kinesiology taping; CI, confidence interval; LB/UB, lower/upper bound.

### Inferential statistics

Data for mean (*M*) ± standard deviation (*SD*) are presented in Table 2. Mixed ANOVA applied to IR and VR datasets found no statistically significant main effect in mean blood perfusion units for Intervention [IR (*F*_2, 42_ = .27, *p* = .766, η_p_^2^ = .01), VR (*F*_2, 42_ = .80, *p* = .455, η_p_^2^ = .04). There was no statistically significant interaction between Intervention and Time [IR (*F*_6.449, 135.426_ = 1.35, *p* = .24, η_p_^2^ = .06), VR (*F*_5.734, 120.407_ = 1.31, *p* = .260, η_p_^2^ = .059)]. There was a statistically significant main effect for Time [IR (*F*_3.224, 135.426_ = 40.46, *p* = .00, η_p_^2^ = .49), VR (*F*_2.867, 120.407_ = 46.81, *p* ≤ .0005, η_p_^2^ = .527)]. Bonferroni post-hoc test results showed that blood perfusion units were statistically significantly decreased at all time points when compared with pre-intervention for IR and VR datasets. There was a marginal statistical non-significant increase in blood perfusion from during-intervention-20-mins to post-intervention (Fig 5) (see S1 Table for pairwise comparisons between all time points).

## Discussion

The purpose of this study was to evaluate the elastic properties of kinesiology tape on cutaneous microcirculation by applying kinesiology tape at approximately 40% stretch using the ‘space correction method’ to lift the skin to create a ‘pocket of space’ between the two ends of the tape. The study found no statistically significant differences in cutaneous blood perfusion between kinesiology taping and standard taping, nor between the taping and no tape interventions when measured using LDI at IR and VR wavelengths in healthy participants at resting conditions. There were significant decreases in blood perfusion over the time course of the experiment across the whole sample.

These findings are consistent with previous such studies conducted in healthy human adults [27–29]. Miller et al. [27] found significant reductions in cutaneous blood flow over the biceps brachii with kinesiology tape applied with 15–20% stretch and athletic tape (no stretch) in a randomised crossover study using 10 participants. Woodward et al. [28] found no significant differences in forearm skin blood flow after 5–10 minutes of local heat provocation between kinesiology taping (techniques described by Kenzo Kase, approximately 35% stretch) and no taping control in a randomised crossover study using 13 healthy participants. Stedge et al. [29] found no significant differences in gastrocnemius muscle blood flow between kinesiology taping (techniques described by Kenzo Kase), sham taping (single strip of kinesiology tape over muscle belly) and no taping controls in a randomised controlled parallel group study using 61 healthy participants who had performed 30 concentric plantar-flexion and dorsiflexion exercises on each test day.

It is necessary to consider the possibility that the finding that there were no statistically significant differences in blood perfusion between kinesiology taping and standard taping, nor between the taping and no tape interventions is due to a lack of sensitivity of the experimental assay (i.e., a false negative finding). Extraneous variables that may have influenced cutaneous microvascular function, which were not taken into consideration in the analyses include sex, age, ethnicity, dietary intake and diurnal variations [45,61–64]. In addition, some participants may have exercise-induced cutaneous microvascular adaptations in their dominant hands, in whom the within-subject change in blood perfusion through the course of repeated measures would have been intrinsically different to those in whom non-dominant hand was assessed [65–68]. It is plausible that these factors contributed to ‘noise’ in data that could conceal the presence of a true physiological change (signal) associated with the intervention(s), i.e., the experimental assay did not have sufficient sensitivity to detect the presence of intervention(s) effects. This seems unlikely because the statistical analyses were able to detect a decline in blood perfusion in all participants across the course of the experiment, i.e., main effects associated with Time, until time point during-intervention-20-mins. The possibility that this drift in blood perfusion measurements could have generated experimental noise in the data that could have swamped the effect of the intervention (i.e., the signal) seems unlikely because the analyses detected a transient increase in blood perfusion between during-intervention-20-mins and post-intervention measurements in participants who were allocated to the taping interventions; this is likely to have been due to the skin irritation caused by tape removal. In participants who did not receive a taping intervention, the blood perfusion declined steadily through the course of the experiment.

The possibility that the decline in blood perfusion over the course of the ‘time-locked experiment’ was a systematic error cannot be discounted and merits explanation. Variations in skin temperature and blood pressure can affect blood perfusion [30,45,69]. The laboratory was maintained at a neutral temperature (normothermic environment) and blood perfusion measurements were taken in resting conditions, although skin temperature and blood pressure were not recorded throughout the experiment and therefore could be a potential source of systematic error in LDI data. In a normothermic environment, cutaneous blood microcirculation at physiologically steady-state condition is approximately 250 ml/min [13], and unless there is a physiological need to increase blood flow (for e.g., rise in body/core temperature caused by physical exercise that is of sufficient intensity and duration) cutaneous microcirculation changes little [68]. The 15-minute pre-experiment acclimatisation should have ensured that participants had achieved a rested state prior to the start of measurements with blood perfusion stabilised. Thus, the most likely explanation for the decline in blood perfusion over the time course of the experiment is an increasing state of relaxation caused by prolonged sitting with minimal movement in a comfortable normothermic environment. It might be necessary to provoke increases in baseline microcirculation in order to observe changes associated with taping.

The possibility that sub-optimal taping technique contributed to the absence of effect needs consideration. The kinesiology taping technique employed in this study was based on its principles and practice as described by Kase et al. [56], it is possible that other methods of taping described by educators of taping may produce different results. It was notable that convolutions of the skin were not visible in some participants in the kinesiology taping group while skin convolutions were noticeable in some participants in the standard taping. This was despite consistent application techniques across the sample. Interestingly, the investigator documented in case report forms observations that suggested that White participants were more likely to have visible taping-induced skin convolutions than participants from other ethnic backgrounds. Whether morphological and functional differences in the human skin associated with ethnicity and age [70] influence response to taping remain unknown. Nevertheless, the findings of the study suggest that convolutions did not influence microcirculation to any appreciable extent.

The findings of this study and previous experimental studies conducted in healthy human adults [27–29] challenge claim about the ‘microcirculatory’ mechanism of action of kinesiology taping. Claims that ‘skin-lifting’ associated with kinesiology taping facilitates the flow of lymph and venous blood appears paradoxical to the conventional wisdom of using compression therapies to manage lymphoedema [71] and venous insufficiency [72]. Future research could use non-invasive imaging techniques such as ultrasonography and Doppler imaging to scrutinise whether mechanical deformations produced in epidermal and dermal layers take place during kinesiology taping and their effect on the microcirculation. In addition, it may be useful to investigate whether kinesiology taping affects cutaneous blood microcirculation in healthy adults with raised or lowered baseline microcirculation, and under exercise testing conditions where there is a physiological need to recruit otherwise constricted capillaries for increasing blood flow to meet metabolic demand and dissipate heat [73]. The findings from previous studies in healthy participants suggest not, although replication of these studies using improvements in methodology and more sensitive imaging techniques (for e.g., capillary microscopy and near infrared spectroscopy) would be useful. Whether kinesiology taping affects cutaneous blood microcirculation in the presence of pathophysiology remains a matter for debate. Evidence from animal models and clinical studies in humans with blood and lymphatic microcirculatory dysfunction is insufficient and inconclusive, although the systematic reviews [6–9] suggest potential benefit for cancer-related lymphedema. Hence, there appears merit in further investigation of kinesiology taping in clinical populations with microcirculatory dysfunction via a large scale robust RCT to determine efficacy.

One of the strengths of this study is that it addressed the shortcoming related to imaging in previous studies (that used LDF) by using dual wavelength LDI to assess blood perfusion in superficial and deeper skin regions. However, the present study was not controlled for variations in extraneous variables including skin temperature and blood pressure, which can influence cutaneous blood perfusion imaging. The lack of adequate research therapist as well as assessor and participant blinding is a notable methodological limitation. It is, however, argued that the impact of the lack of participant blinding on the results of this study would be minimal as the participants were naïve to the study hypothesis and that the primary outcome was measured objectively. The findings of this study must be interpreted with caution because of the low sample size, and are not generalisable to clinical practice in patients with microcirculatory dysfunction as the study was conducted using a sample of healthy human participants in resting conditions.

## Conclusions

In conclusion, the findings of this study suggest that kinesiology taping does not increase cutaneous blood flow in healthy human adults under resting physiological conditions in a normothermic environment. It appears that the use of kinesiology taping intended to provide therapeutic benefits from improved blood microcirculation in the absence of microcirculatory dysfunction is unwarranted.

## Supporting information

S1 Fig Figure showing screenshot of sample size estimation in G*power software. (TIF)

S1 Table Pairwise comparisons between all time points for (log10 transformed) infrared (IR) and visible-red (VR) data. Bonferroni correction for multiple comparisons. *Mean difference statistically significant at < 0.05.
(DOCX)

S1 Data (XLSX)

S2 Data (XLSX)
