# Supplementary figures and images for: The immediate effects of kinesiology taping on cutaneous blood flow in healthy humans under resting conditions: A randomised controlled repeated-measures laboratory study
Source: PLoS One. 2020 Feb 21;15(2):e0229386. doi: 10.1371/journal.pone.0229386 (PMC7034885; doi:10.1371/journal.pone.0229386)

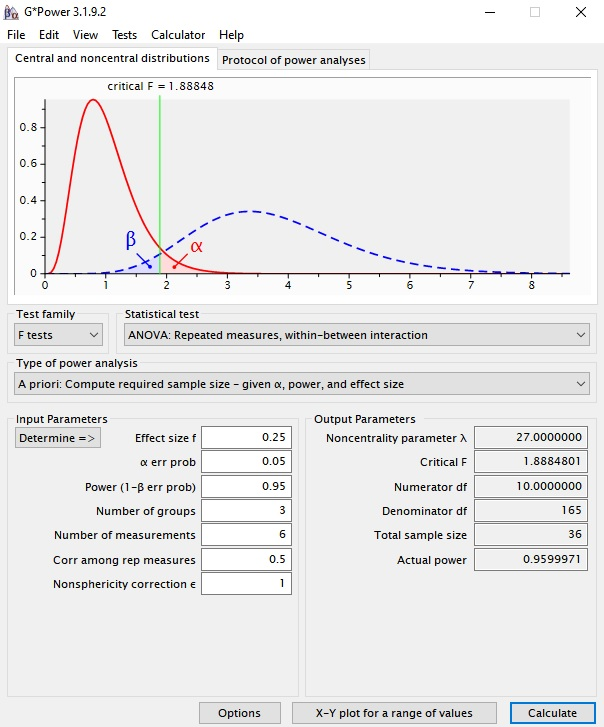

Supplement: S1 Fig — (TIF) [file pone.0229386.s001.tif]
