# Supplementary material for: The immediate effects of kinesiology taping on cutaneous blood flow in healthy humans under resting conditions: A randomised controlled repeated-measures laboratory study
Source: PLoS One. 2020 Feb 21;15(2):e0229386. doi: 10.1371/journal.pone.0229386 (PMC7034885; doi:10.1371/journal.pone.0229386)
Supplement: S1 Table — Bonferroni correction for multiple comparisons. *Mean difference statistically significant at < 0.05. (DOCX) [file pone.0229386.s002.docx]

| Time points | | IR p-value* | VR p-value* |
| --- | --- | --- | --- |
|  |  |  |  |
| Pre-intervention | During- intervention-5-mins | .000 | .000 |
|  | During--intervention-10-mins | .000 | .000 |
|  | During- intervention-15-mins | .000 | .000 |
|  | During- intervention-20-mins | .000 | .000 |
|  | Post- intervention | .000 | .000 |
| During-intervention-5-mins | Pre- intervention | .000 | .000 |
|  | During--intervention-10-mins | 1.000 | 1.000 |
|  | During- intervention-15-mins | .000 | .000 |
|  | During- intervention-20-mins | .003 | .000 |
|  | Post- intervention | .178 | .006 |
| During-intervention-10-mins | Pre- intervention | .000 | .000 |
|  | During- intervention-5-mins | 1.000 | 1.000 |
|  | During- intervention-15-mins | .081 | .004 |
|  | During- intervention-20-mins | .051 | .000 |
|  | Post- intervention | 1.000 | .054 |
| During-intervention-15-mins | Pre- intervention | .000 | .000 |
|  | During- intervention-5-mins | .000 | .000 |
|  | During- intervention-10-mins | .081 | .004 |
|  | During- intervention-20-mins | 1.000 | .404 |
|  | Post- intervention | 1.000 | 1.000 |
| During-intervention-20-mins | Pre- intervention | .000 | .000 |
|  | During- intervention-5-mins | .003 | .000 |
|  | During--intervention-10-mins | .051 | .000 |
|  | During- intervention-15-mins | 1.000 | .404 |
|  | Post- intervention | 1.000 | 1.000 |
| Post-intervention | Pre- intervention | .000 | .000 |
|  | During- intervention-5-mins | .178 | .006 |
|  | During--intervention-10-mins | 1.000 | .054 |
|  | During- intervention-15-mins | 1.000 | 1.000 |
|  | During- intervention-20-mins | 1.000 | 1.000 |

Table S1. Pairwise comparisons between all time points for (log10 transformed) infrared (IR) and visible-red (VR) data. Bonferroni correction for multiple comparisons. *Mean difference statistically significant at < 0.05
